# Editorial to “Novel mapping techniques for ablation of non‐pulmonary vein foci using complex signal identification”

**DOI:** 10.1002/joa3.70006

**Published:** 2025-01-23

**Authors:** Yoshiaki Mizutani, Satoshi Yanagisawa, Yasuya Inden

**Affiliations:** ^1^ Department of Cardiology Yokkaichi Municipal Hospital Yokkaichi Mie Japan; ^2^ Department of Cardiology Nagoya University Graduate School of Medicine Nagoya Aichi Japan

A mechanism of paroxysmal atrial fibrillation (AF) involves trigger activity mainly originating from a pulmonary vein (PV). Catheter ablation of PV isolation, using recent advanced technologies, is a promising approach to prevent AF incidence and related complications. However, some AF triggers originate from non‐PV foci, which are associated with AF recurrence despite complete PV isolation.^1^, ^2^ Although various approaches and techniques have been introduced for induction and provocation of non‐PV foci, identifying the exact location of non‐PV foci in the broad area of the left and right atriums is still challenging. Fractionated signal area in the atrial muscle (FAAM) map‐guided ablation is a recently developed technique that highlights the fractionated signal area using the LUMIPOINT software in the ultrahigh‐density RHYTHMIA mapping system (Boston Scientific, Marlborough, MA). These fractionated signal areas are significantly associated with the location of non‐PV foci.^3^ The FAAM‐guided ablation previously demonstrated a lower recurrence rate of atrial tachyarrhythmia compared to the non‐FAAM ablation in patients with recurrent AF who underwent catheter ablation targeting non‐PV foci.^3^ Unfortunately, this specific FAAM map can only be used in the RHYTHMIA mapping system, underscoring the need for broader utility of this algorithm across all mapping systems in clinical practice.

In this issue of the *Journal of arrhythmia*, Kono. et al.^1^ reported a successful non‐PV foci ablation case for paroxysmal AF using a Complex Signal Identification (CSI) algorithm equipped with CARTO™ 3 system version 8 to automatically identify and tag complex fractionated potentials in atria. After PV isolation and cavotricuspid isthmus ablation, an additional ablation was performed using the CSI algorithm to target non‐PV foci triggered by isoproterenol infusion, high‐rate burst pacing, and adenosine triphosphate administration. High CSI tag scores were found in the anterior carina of the right superior PV (RSPV), extending to the anterior wall. The earliest activation site in the non‐PV‐foci corresponded to the highest CSI score of 9.8, with fractionated potentials where effective energization was applied. Additionally, the PV isolation line for the right superior PV was slightly extended to include the high CSI area of ≥7.5. At the end of the ablation, no AF was induced, and the patient maintained sinus rhythm without antiarrhythmic drugs for 6 months.

The CSI algorithm can arbitrarily calculate the abnormal potentials using four parameters: minimum fractionated score, time frame within the window of interest, bipolar amplitude of the complex signal, and minimum duration, implying a strict stratification for the relevant fractionated potentials from broad perspectives. Unfortunately, appropriate CSI setting and cutoff points have not been established, and each individual case requires its own CSI setting and score, although the present case adopted a cut‐off point of CSI with 7.5 to elucidate the significant fractionated potentials potentially associated with the non‐PV foci. Furthermore, the CSI score could change with heart rate, rhythm type, and pacing site. It would be interesting to assess whether the result in the present cases would be the same among multiple pacing sites and different pacing rates. It would also be interesting to see if a high CSI score was present in the right atrium which is a typical origin of non‐PV foci, or on the opposite side of the septum from the left atrium, although the non‐PV foci no longer appeared after the left atrium septal ablation. In addition, it should be noted that the internal algorithm and mathematical calculation to classify the fractionated potentials are not the same between the CSI and FAAM modules, raising a cautionary note for using the CSI system for searching non‐PV foci, similar to the FAAM mapping system. Thus, further clinical research with adequate sample size is required to validate this CSI module as a selective ablation strategy targeting non‐PV foci and to improve clinical outcomes and prognosis.

## FUNDING INFORMATION

This research did not receive any specific grants from funding agencies in the public, commercial, or not‐for‐profit sectors.

## CONFLICT OF INTEREST STATEMENT

The authors declare no conflicts of interest.
